# Traumatic Brain Injury and Subsequent Dementia in a Longitudinal Cohort of Older Adults

**DOI:** 10.1002/alz70860_103817

**Published:** 2025-12-23

**Authors:** John H Kanter, L. Grisell Diaz‐Ramirez, Kristine Yaffe, Raquel C. Gardner, W John Boscardin, Michele Diaz, Erica S Kornblith

**Affiliations:** ^1^ University of California, San Francisco, San Francisco, CA, USA; ^2^ University of California San Francisco / San Francisco VA Medical Center, San Francisco, CA, USA; ^3^ NCIRE‐The Veterans Health Research Institute, San Francisco, CA, USA; ^4^ Sheba Medical Center, Joseph Sagol Neuroscience Center, Ramat Gan, Israel; ^5^ San Francisco Veterans Affairs Health Care System, San Francisco, CA, USA; ^6^ University of California San Francisco and Zuckerberg San Francisco General Hospital, San Francisco, CA, USA

## Abstract

**Background:**

This study aimed to ascertain the association between incident traumatic brain injury (TBI) and cognitive outcomes in older adults (OA), comparing development of dementia (DR) over time in OA with and without TBI using the longitudinal, nationally representative Health and Retirement Study (HRS) dataset.

**Method:**

Community‐dwelling OA aged 65+ with incident TBI, identified through Medicare diagnosis codes in inpatient and outpatient files (2000 to 2018), were included. TBI respondents were matched 1:1 to non‐TBI controls by demographic factors (age, gender, race/ethnicity), pre‐ HRS interview variables [HRS wave, mode of interview (i.e., in‐person vs. remote), education, and wealth]. Analyses included Fine and Gray competing risk regression models. Dementia was defined using the algorithmic Hudomiet latent cognitive ability approach, with Medicare dementia codes used as an alternate dementia outcome in sensitivity analyses.

**Result:**

This retrospective cohort included 1576 community‐dwelling OA, 363 (22.4%) of whom developed dementia during the 16‐year study follow‐up period. Unadjusted models indicated that participants with TBI had a roughly 60 percent increase in the subdistribution hazard of dementia (sHR = 1.57, 95% CI: 1.28‐1.93). Imputed Fine and Grey competing risk regression models fully adjusted for medical and psychiatric covariates showed a similarly increased subdistribution hazard (sHR = 1.50, 95% CI: 1.21‐1.85). In sensitivity analyses using Medicare dementia codes, the increased subdistribution hazard likewise persisted in both unadjusted (sHR = 1.513, 95% CI: 1.191‐1.922) and imputed, fully adjusted (sHR = 1.512, 95% CI: 1.191‐1.921) models.

**Conclusion:**

Incident TBI in OA may be associated with higher risk of subsequently developing dementia compared to non‐TBI counterparts. These results emphasize the importance of early cognitive screening and potential interventions for OA with TBI. Further research should explore mechanisms driving cognitive decline post‐TBI and how socioeconomic and behavioral factors may impact these outcomes.

